# Genomic Characterization of *Listeria innocua* Isolates Recovered from Cattle Farms, Beef Abattoirs, and Retail Outlets in Gauteng Province, South Africa

**DOI:** 10.3390/pathogens12081062

**Published:** 2023-08-18

**Authors:** James Gana, Nomakorinte Gcebe, Rian Ewald Pierneef, Yi Chen, Rebone Moerane, Abiodun Adewale Adesiyun

**Affiliations:** 1 Department of Production Animal Studies, Faculty of Veterinary Science, University of Pretoria, Onderstepoort 0110, South Africa; jamesgana38@gmail.com (J.G.); rebone.moerane@up.ac.za (R.M.); 2 Agricultural Education, Federal College of Education, Kontagora 923101, Nigeria; 3 Bacteriology Department, Onderstepoort Veterinary Research, Agricultural Research Council, Pretoria 0110, South Africa; gceben@arc.agric.za; 4 Department of Biochemistry, Genetics and Microbiology, University of Pretoria, Pretoria 0001, South Africa; rian.pierneef@up.ac.za; 5 Centre for Bioinformatics and Computational Biology, University of Pretoria, Pretoria 0001, South Africa; 6 Microbiome@UP, Department of Biochemistry, Genetics, and Microbiology, University of Pretoria, Pretoria 0001, South Africa; 7 Center for Food Safety and Applied Nutrition, US Food and Drug Administration, 5001 Campus Dr. Room 4E-007/Mailstop HFS-710, College Park, MD 20740, USA; yi.chen@fda.hhs.gov; 8 School of Veterinary Medicine, Faculty of Medical Sciences, University of the West Indies, St. Augustine 685509, Trinidad and Tobago

**Keywords:** beef production chain, *Listeria innocua*, whole-genome sequencing, sequence type, antimicrobial resistance genes, virulence gene, South Africa

## Abstract

Whole-genome sequencing (WGS) was used for the genomic characterization of one hundred and ten strains of *Listeria innocua* (*L. innocua*) isolated from twenty-three cattle farms, eight beef abattoirs, and forty-eight retail outlets in Gauteng province, South Africa. In silico multilocus sequence typing (MLST) was used to identify the isolates’ sequence types (STs). BLAST-based analyses were used to identify antimicrobial and virulence genes. The study also linked the detection of the genes to the origin (industries and types of samples) of the *L. innocua* isolates. The study detected 14 STs, 13 resistance genes, and 23 virulence genes. Of the 14 STs detected, ST637 (26.4%), ST448 (20%), 537 (13.6%), and 1085 (12.7%) were predominant, and the frequency varied significantly (*p* < 0.05). All 110 isolates of *L. innocua* were carriers of one or more antimicrobial resistance genes, with resistance genes *lin* (100%), *fosX* (100%), and *tet*(*M*) (30%) being the most frequently detected (*p* < 0.05). Of the 23 virulence genes recognized, 13 (*clpC*, *clpE*, *clpP*, *hbp1*, *svpA*, *hbp2*, *iap/cwhA*, *lap*, *lpeA*, *lplA1*, *lspA*, *oatA*, *pdgA*, and *prsA2*) were found in all 110 isolates of *L. innocua*. Overall, diversity and significant differences were detected in the frequencies of STs, resistance, and virulence genes according to the origins (source and sample type) of the *L. innocua* isolates. This, being the first genomic characterization of *L. innocua* recovered from the three levels/industries (farm, abattoir, and retail) of the beef production system in South Africa, provides data on the organism’s distribution and potential food safety implications.

## 1. Introduction

*Listeria* species consist of a group of non-spore-forming Gram-positive facultative anaerobic coccobacilli [1]. There are 21 species of *Listeria* documented since 2020, but few are known to be pathogenic to animals and/or humans [2]. *Listeria monocytogenes* (*L. monocytogenes*) is the only recognized human pathogen and is also pathogenic to animals [3,4]. After consumption, several contaminated food types, such as milk and milk products, vegetables, meat, and meat products, have been implicated in listeriosis sporadic cases and/or outbreaks [5,6]. Some clinical manifestations of listeriosis in humans include fever, muscle aches, nausea, vomiting, stomach cramps, diarrhea, abortion, preterm birth, stillbirth in pregnant women, meningitis or encephalitis, and death [7,8].

Currently, *Listeria ivanovii* is known to cause listeriosis in animals [2]. Although rare cases of human and animal infections were reported [9,10], *L. innocua* is not recognized as a human pathogen. *Listeria innocua* (*L. innocua*) co-exists in the same food and environmental niches as *L. monocytogenes*, thus serving as indicator organisms for *L. monocytogenes* [11,12].

*L. innocua* has been documented to share some virulence factors with *L. monocyte-genes* [13], including some *Listeria* pathogenic islands (*Listeria* pathogenic island (LIPI)-1, LIPI-3, and LIPI-4) [14,15]. Virulence genes contribute to the virulence and pathogenicity of *L. monocytogenes.* Several virulence genes have been reported in strains of *L. innocua*, but the prevalence and roles of these virulence genes have not been elucidated [16].

Classical multi-locus sequence typing (MLST) targeting six to eight housekeeping/virulence genes and core genome-based (cg) MLST genotyping have been essential in the epidemiological investigation and surveillance of *L. monocytogenes* isolates [17]. Numerous Sequence Types (STs) have been documented, mainly for *L. monocytogenes*. Since these housekeeping genes are also present in other *Listeria* spp., STs have also been assigned to *L. innocua* and other *Listeria* spp. [18,19]. Furthermore, some STs of *L. monocytogenes* have been associated with human listeriosis, and their occurrence and distribution are affected by geographical locations and food types [20,21].

Strain typing techniques such as traditional serotyping, multi-virulence-locus sequence typing (MVLST), MLST, multilocus variable number tandem repeat analysis (MLVA), and pulse-field gel electrophoresis (PFGE), among others, have been used to detect and characterize *Listeria* spp. [22]. However, whole-genome sequencing (WGS) and various in silico analyses based on WGS are currently the methods of choice for molecular sub-typing for *Listeria* spp. as they provide a higher resolution of strains than the other methods [23,24].

The use and abuse of antimicrobial agents in humans and animals have resulted in the expansion of bacterial antimicrobial resistance, and some bacteria are resistant to multiple antimicrobials [25]. The genes that encode antimicrobial resistance (AMR) in *Listeria* spp. are well documented [19,26]. Still, it is also known that the resistance genes carried by bacteria may not be expressed, limiting their importance in assessing their clinical or therapeutic significance [27]. Variable frequencies of AMR genes have been reported in strains of *L. monocytogenes*, *L. innocua*, and other *Listeria* spp. [28,29]. Hosain et al. [30] have highlighted the potential negative impact of AMR on therapy in feed animals and humans. To date, there has not been any information on the genomic carriage of resistance genes by *L. innocua* in South Africa.

Between 2017 and 2018, South Africa experienced the world’s largest outbreak of human listeriosis, caused by ‘polony’, a ready-to-eat (RTE) beef product [31], and *L. monocytogenes* ST6 was determined to be the etiological agent [21]. A few studies using polymerase chain reaction (PCR) have characterized *L. monocytogenes* strains recovered from meat and meat products across the country [32] and MLVA genotypes of *Listeria monocytogenes* and *L. innocua* isolated from farms, abattoirs, and retail in Gauteng province [33], Mpumalanga province, and North–West province [34]. To the best of our knowledge, there is a dearth of information on the genomic characteristics of *L. innocua* strains in the country. Mafuna et al. [35] used WGS to characterize 38 isolates of *L. innocua* recovered from the country’s meat and food processing facilities, while ElZowalaty et al. [36] reported the genomic sequence of one strain of *L. innocua* isolated from a healthy goat. The genomic characteristics of *L. innocua* at different levels of the beef production chain are currently unknown.

Therefore, the specific objectives of this study were to use WGS to characterize strains of *L. innocua* isolates from samples collected from cattle farms, beef abattoirs, and retail outlets in Gauteng, South Africa, to elucidate the diversity in the profiles of their sequence types, resistance genes, and virulence genes. The study also investigated the relationships between the profiles and the sources and sample types from which the isolates originated.

## 2. Materials and Methods

### 2.1. Source of L. innocua Used in the Current Study

The isolates of *L. innocua* subjected to WGS in the current study originated from an earlier study. Details on the types and number of samples collected from the sources mentioned earlier and the types of samples in the current study have been documented [37].

### 2.2. Study Design and Sources of Samples

**Figure 1:**
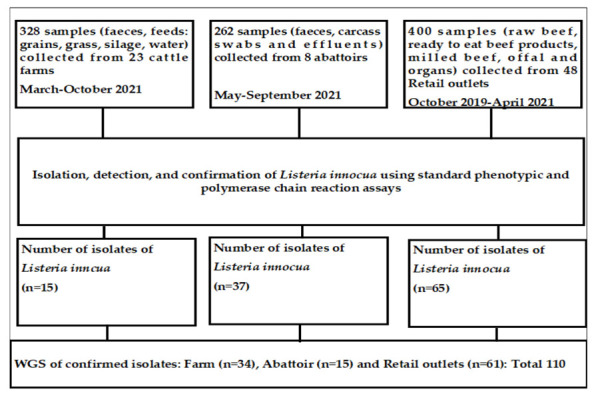
Schematic diagram of the study design using three cross-sectional studies.

In South Africa, three major industries constitute beef production, processing, and retailing, namely the cattle farm, abattoir, and retail industries. Therefore, three cross-sectional studies were conducted in three industries in Gauteng province. Figure 1 provides details on the types and number of samples collected. The sample size was determined as recommended by Thrusfield [38].

### 2.3. Variables of Beef Industries Investigated in the Cross-Sectional Studies

#### 2.3.1. Cattle Farms

In South Africa, three categories of cattle farms are used in cattle production: communal farms, cow-calf operations, and feedlots.

i. Communal farms: South Africa has about 18 million hectares of communal land. This is owned by the government but managed by tribal authorities. Livestock owned by several owners graze collectively in the community and are taken to vaccination and ectoparasite control centers in the area as a large herd (owned by many farmers) for vector control. Ten communal farms were sampled for the current study.
ii. Cow-calf operations: These operations refer to farms that breed and raise cattle to sell them. These farmers are focused on raising quality cattle that are suitable for the specific industry they sell them to, such as dairy cattle or beef cattle. A total of 10 cow-calf operations were included in the current study.
iii. Feedlots: Feedlots purchase cattle and prepare them for the final stage of the beef production process. Feedlots are focused on feeding mature cattle and ensuring that they have the right medical clearance to continue the beef production process. The input of private veterinary services is optimal at the feedlots, and the majority have their own abattoirs. Samples for the current study were collected from three feedlots. In South Africa, feedlots contribute 60–65% of the cattle slaughtered.

#### 2.3.2. Abattoirs

Cattle are slaughtered at three different venues to obtain beef in the country: butcheries, low-throughput abattoirs, and high-throughput abattoirs.

i. Butcheries: These are small operations by individuals where cattle are slaughtered primarily from small farms (communal and cow-calf operations) and the beef is sold fresh on-site to the consumers. The animals slaughtered at these facilities are not inspected by the veterinary public health (VPH) personnel, either pre-slaughter or post-slaughter. The samples originating from these slaughter facilities are mostly sold directly to consumers.
ii. Low-throughput (LT): The facilities are so classified by the veterinary public health section of the Department of Agriculture, Forestry, and Fisheries (DAFF). In the country, all abattoirs are privately owned, but all animals slaughtered at these facilities are legally expected to be inspected before and after slaughter by VPH personnel. LT abattoirs slaughter livestock, including red-meat livestock such as sheep, pigs, and goats. Game and poultry are slaughtered at different abattoirs in the country. LT abattoirs are classified based on the maximum daily throughput of livestock, ranging from 20 units for cattle, 30 for pigs, and 40 for sheep and goats if only one species is slaughtered (Red Meat Regulation R1072 from Meat Safety Act 40 of 2000) [39]. Cattle slaughtered at LT abattoirs primarily originate from communal farms and cow-calf operations. For our study, eight LT abattoirs were randomly selected for sampling where cattle were slaughtered.
iii. High-throughput (HT): The activities that take place at the HT abattoirs are similar to those at the LT abattoirs, except for the fact that they have a higher maximum daily throughput that is determined by the provincial executive officer on the grounds of lairage capacity and hourly throughput potential relative to available equipment and infrastructure, as stated by the Red Meat Regulation R1072 from Meat Safety Act 40 of 2000 [39]. HT abattoirs are classified based on units of daily slaughter exceeding those stated for LT abattoirs, as stated above. Most feedlots have their own individual HT abattoirs. A total of six HT abattoirs were sampled in the current study.

#### 2.3.3. Retail Outlets

A total of 48 retail outlets located in various districts in Gauteng province were randomly selected to serve as retail industry sources of beef and beef products. The retail outlets were classified using their distribution and size, estimated by the number of cashiers. The four types of retail outlets that served as sources of the samples were as follows:

i. Chain retail outlets: These are retail outlets with two or more outlets distributed across the province. Samples were collected from 30 chain retail outlets.
ii. Large retail outlets: Outlets with six or more cashiers, from which 10 were recruited for sampling.
iii. Medium retail outlets: These are outlets with 3–5 cashiers with sampling from 6 outlets.
iv. Small retail outlets: Outlets with 1–2 cashiers, from which two outlets were sampled.

### 2.4. Variables of Sample Types Investigated in the Cross-Sectional Studies and Investigating the Potential Effect of the Source of Samples on the Distribution and the Characteristics of L. innocua

The sample types collected from the cattle farms, abattoirs, and retail outlets are shown in Figure 1. Regardless of the types of samples collected at the three beef industries (cattle farms, abattoirs, and retail outlets), and although the investigation involved three cross-sectional studies and not a longitudinal study, the rationale was to determine the genomic relationship of *L. innocua* isolates (AMR genes and virulence genes) recovered from the three industries. This is because of the potential transfer of *L. innocua* from cattle farms to the abattoirs where they are slaughtered and finally to the retail outlets from where the beef and beef products get to the consumer, thus having epidemiological significance.

### 2.5. Isolation, Identification of L. innocua, and Determination of AMR

All 110 isolates of *L. innocua* were previously identified (bacteriological and multiplex PCR) as *L. innocua* as described [40,41]. The confirmed isolates of *L. innocua* were inoculated in 50% brain heart infusion (BHI)/50% glycerol and stored at −20 °C until subjected to whole genome sequencing (WGS) analyses. The number of isolates of *L. innocua* used in this study was 110 (11.1%) from 990 samples. The prevalence of *L. innocua* in the three beef industries was 10.4% (34/328), 5.7% (15/262), and 15.3% (61/400) for cattle farms, beef abattoirs, and retail outlets, respectively. The current study assessed all the isolates, their origin (three industries: cattle farms, abattoirs, and retail), and the types of samples.

### 2.6. Selection of Antimicrobial Agents Used and Determination of the Resistance of Listeria Isolates to Antimicrobial Agents

For this study, 16 antimicrobial agents were used, and the selection was based on their ease of availability to livestock farmers, their use by veterinary and medical practitioners, and feedback received following consultation with veterinarians in Gauteng province, South Africa. The types and disc concentrations of antimicrobial agents (Thermo Fisher Scientific, Germiston City, South Africa) used were as follows: Penicillin (10 units), Amoxicillin clavulanic acid (30 μg), Ampicillin (10 μg), Cephalothin (30 μg), Cefotaxime (30 μg), Streptomycin (25 μg), Gentamicin (10 μg), Kanamycin (30 μg), Tetracycline (30 μg), Doxycycline (30 μg), Nalidixic acid (30 μg), Ciprofloxacin (5 μg), Enrofloxacin (5 μg), Azithromycin (15 μg), Clindamycin (10 μg), and Sulfamethoxazole-Trimethoprim (23.75/1.25 μg).

To determine the resistance of *Listeria* spp. to 16 antimicrobial agents among the isolates of *Listeria* spp., the Kirby-Bauer disk diffusion method according to the description and the interpretation criteria recommended by the Clinical and Laboratory Standards Institute [42] was used. For the antimicrobial agents for which the cut-off values for susceptibility were not stated for *Listeria*, the values provided for staphylococci were used as earlier recommended [43]. The following strains were used as controls: *L. monocytogenes* ATCC 19111, *Listeria innocua* ATCC 33090, *L. welshimeri* ATCC 35897, and *Campylobacter fetus* ATCC 27373. For this study, any isolate that exhibited intermediate (I) or resistance (R) was classified as resistant to the antimicrobial agent.

### 2.7. Whole-Genome Sequencing, Genomic Analysis, Assembly, and Annotation

DNA extraction was performed using the Qiagen DNAEasy Blood & Tissue kit, manual, Gram-positive protocol, as per the manufacturer’s instructions. All isolates were sequenced on an Illumina MiSeq platform (250-bp paired-end reads; Illumina, Inc., San Diego, CA, USA) using the Nextera XT library preparation kit per the manufacturer’s instructions.

Quality control, including adapter removal of the raw data, was conducted using BBDuk (v.38.91; https://jgi.doe.gov/data-and-tools/bbtools/bb-tools-user-guide/bbduk-uide/; sourceforge.net/projects/bbmap/).(accessed on 6 September 2022) SPAdes v.3.15.3 [44] created a de novo assembly of each isolate. Only contigs longer than 500 bp were retained for further analysis. Completeness and contamination of the de novo assemblies were assessed with CheckM v.1.1.3 [45], and taxonomic classification was performed using GTDB-Tk v.1.7.0 [46]. The details have been provided in Supplementary Data, Table S1.

### 2.8. In Silico MLST

MLST STs were determined using the MLST tool [47], which makes use of the PubMLST website (https://pubmlst.org/) developed by Keith Jolley (Jolley & Maiden 2010, BMC Bioinformatics, 11:595) and sited at the University of Oxford. The development of that website was funded by the Wellcome Trust. The latest *Listeria* ST scheme was obtained from BIGSdb-Lm (accessed 21 July 2023) [48] and incorporated into the MLST tool.

### 2.9. Resistance and Virulence Profiles

ABRicate [49] was used to detect antimicrobial resistance genes and virulence factors in species of interest. Abricate was run with default parameters, and the NCBI database was selected for AMR detection. This database was locally updated on 2 November 2022 and, at the time of usage, included 6334 sequences (doi: 10.1128/AAC.00483-19). For virulence factors, the “vfdb” database was used, updated on 2 November 2022, and containing 4332 sequences (doi: 10.1093/nar/gkv1239).

### 2.10. Construction of the Phylogenetic Tree for L. innocua Isolates and Correlation with Source and Type of Samples

A multiple protein sequence alignment was constructed using GTDB-Tk v.1.7.0 [50] and based on 120 GTDB core bacterial marker genes. FastTree v.2.1.11 was used to infer a phylogenetic tree and visualized in R with ggtree [51].

### 2.11. Data Analysis

All data analyses were performed using R v.4.1.2 [52], implemented In RStudio v.2022.2.3.492 [53]. Distance matrices were calculated using the “daisy” function with the “gower” parameter specified to determine Gower distances with the R package “cluster” [54]. Minimum spanning trees were calculated using the “ape” package [55], with the “mst” function, and visualized using “igraph” [56] and “ggnetwork” [57]. R packages ggstatsplot [58], ggsci [59], and ggpubr [55] were further used for data analysis and visualization. The ggstatsplot function, ggscatterstats, was implemented to perform correlation analyses based on Pearson’s correlation coefficient. Pearson’s Chi-squared Test for Count Data, implemented by the chisq.test, was used to test for associations. Pearson’s correlation coefficients were calculated using the cor function. Bar charts were produced using the rm function ggbarstats, and Pearson’s chi-squared test was used to test for significant differences.

## 3. Results

### 3.1. Effect of the Three Beef Industries (Cattle Farms, Abattoirs, and Retail) on the Frequency of Detection of STs, AMR Genes, and Virulence Genes in L. innocua Isolates

The frequency of detection of *L. innocua*, STs, and AMR genes in the isolates is shown in Table 1. The frequency of detection of *L. innocua* by industry varied significantly, ranging from 5.7% (abattoir) to 16.3% (retail). Across three industries, differences were found in the frequencies of four STs: 637, 448, 1537, and 1085. For ST637, the frequency was lowest in abattoirs (13.3%) and the highest in cattle farms (50%); for ST448, the frequency range was from 0% (abattoir) to 32.8% (retail); for ST537, the frequency was lowest (3.3%) in isolates from retail and the highest, 73.3%, in abattoirs; for ST1085, none (0%) of the isolates from abattoirs were positive for the ST, while the highest frequency was detected in retail isolates, 21.3%.

For the AMR genes, the frequency of detection by industry varied significantly (*p* < 0.05) for only the *tet*(*M*) and *dfrG* genes. The lowest frequency of *tet*(*M*) genes was found in farm isolates (20.6%), while the highest was detected in abattoir isolates (73.3%). For the *dfrG* gene, the frequency range was from 0% (cattle farms) to 13.1%. Supplementary data: Table S2 shows the details of the sources, sample types, sequence types, and AMR genes identified in the 110 *L. innocua* isolates.

Twenty-three putative virulence factors (*clpC*, *clpE*, *clpP*, *fbpA*, *gtcA*, *hbp1*, *svpA*, *hbp2*, *iap/cwhA*, *lap*, *llsA*, *llsB*, *llsD*, *llsG*, *llsH*, *llsP*, *llsX*, *llsY*, *lpeA*, *lplA1*, *lspA*, *oatA*, *pdgA*, and *prsA2*) were detected across the 110 isolates of *L. innocua* from the three industries (farms, abattoirs, and retail outlets). Among the 34 isolates from cattle farms, 19 (82.6%) of 23 genes were detected, except for *llsB*, *llsD*, *llsP*, and *llsY*. The frequency of detection of virulence genes ranged from 0% (0/34) to 100% (34/34) in 14 genes. For the 15 abattoir isolates, all 23 (100%) virulence genes were detected, with a range from 6.7% (1/15) for *llsB*, *llsD*, *llsP*, and *llsY* to 100% (15/15) for 15 genes. For the 61 isolates of *L. innocua* from retail outlets assessed for virulence genes, all 23 (100%) genes were detected, and the frequency range was from 8.2% (1/61) for *llsP* to 100% (61/61) in 14 genes.

Overall, the frequency of virulence genes detected across the three industries production ranged from 82.6–100%.

Supplementary data, Table S3, shows the details of the sources, sample types, sequence types, and virulence factor genes identified in the 110 *L. innocua* isolates.

### 3.2. Frequency of STs in Innocua

**Figure 2:**
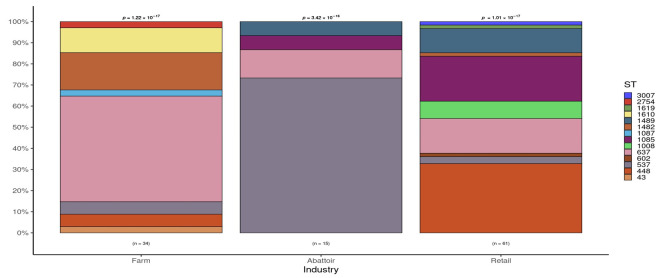
Frequency of *L. innocua* sequence types by industry. Significant associations for each industry based on the ST detected were found, and the associated *p*-values are presented above the bar plot. Overrepresentation of ST637 and ST1482 was seen in the farming industry, whereas ST537 was found to be significantly abundant in the abattoirs. In the retail industry, ST448, ST637, and ST1085 were found more frequently than expected.

Overall, in samples collected from the three beef industry sources (cattle farm, abattoir, and retail), 14 STs were detected in the 110 isolates of *L. innocua* (Figure 2). The frequency of STs found was as follows: ST637 (29 isolates, 26.4%), ST448 (22, 20%), ST537 (15, 13.6%), ST1085 (14, 12.7%), ST1489 (8, 7.3%), ST1482 (7, 6.4%), ST1008 (5, 4.5%), ST1610 (4, 3.6%), and ST1619, ST602, ST1087, ST3007, ST2754, and ST43 (1 isolate each, 0.9%). Proportional testing indicated significant associations for certain STs with each industry (*p* < 0.05). The farming industry displayed an affinity for ST637 and ST1482, abattoirs ST537, and retail ST448, ST637, and ST1085 based on residual values larger than 2.

#### 3.2.1. Minimum Spanning Tree (MST) Based on ST Profiles

**Figure 3:**
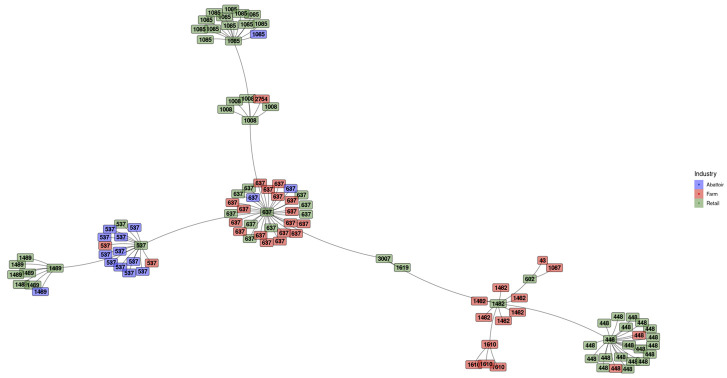
Minimum spanning tree for the sequence types of *L. innocua* isolates colored according to the industry. Homogeneous-colored clusters indicate industries with an affinity for certain STs. In the retail sector, clusters for ST448 and ST1085 are clear, with ST537 regularly found in abattoirs. ST637 occurs frequently in both the farming and retail sectors.

The MST of the STs of *L. innocua* isolates is displayed in Figure 3. A clear clustering of the samples based on the ST profiles was evident. Each isolate is colored according to the industry from which it was obtained. Clusters with color homogeneity indicate industries predisposed to certain STs. In the retail sector, an overrepresentation of ST448 and ST1085 can be seen as a clear group, with ST537 predominantly found in abattoirs. The farming and retail sectors share a high abundance of ST637, as can be seen in the large, multi-colored cluster. Smaller ST-based clusters with industry homogeneity are further evident. The allele scheme for each ST found is available in Supplementary Table S4.

#### 3.2.2. Phylogenies of *L. innocua* Isolates According to the STs and Industry

**Figure 4:**
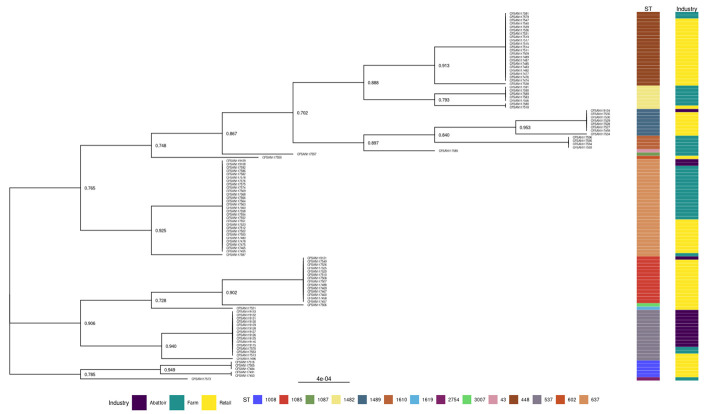
Phylogenetic tree of the 110 recovered isolates across the three industries. The first color map indicates ST, and the second indicates the industry related to the isolate. Bootstrap values are indicated and are high across the tree. Groupings based on ST are evident in the sequential colors in the first color map. associated industry in the second color map indicates a propensity for STs in certain industries.

The genetic relationships of the *L. innocua* isolates recovered from three industries are shown in Figure 4. The tree indicated grouping based on ST, and the high affinity for certain ST in each of the three industries is evident.

### 3.3. Detection of Antimicrobial Resistance Genes in L. innocua

Thirteen resistance genes were detected in the 110 *L. innocua* isolates from the industries tested. They were as follows: *fosX*, one hundred and ten (100%), *lin*, one hundred and ten (100%), *tet*(*M*), thirty-three (30%), *dfrG*, nine (8.2%), *ImuD*, six (5.5%), *mphB*, five (4.5%), *mefA*, four (3.6%), *msrD*, four (3.6%), *tet*(*S*), four (3.6%), *ant.6.1a*, one (0.9%), *InuG*, one (0.9%), *vatB*, one (0.9%), and *vga*, one (0.9%). The details of the sources, sample types, sequence types, and resistance genes identified in the 110 *L. innocua* isolates are shown in Supplementary data: Table S2.

#### 3.3.1. Patterns of Multiple Antimicrobial Resistance Genes

For the 110 isolates of *L. innocua* recovered from farms, abattoirs, and retail outlets, there were ten AMR gene patterns with a range of 2–7 resistance genes per pattern (Table 2). For the thirteen AMR genes detected, the frequency of AMR gene patterns was high for *foxX-lin*, sixty-four (58.2%), *fosX-lin-tetM*, twenty-seven (24.5%), *dfrG-fosX-lin*, and eight (7.3%), but low, one (0.9%), for the other five patterns. The frequency of resistance patterns varied significantly among *L. innocua* collected from cattle farms, abattoirs, and retail outlets.

The frequency of AMR gene patterns varied across the isolates from the three sources (cattle farms, abattoirs, and retail outlets) for *fosX-lin*, *fos-lin-tet*(*M*), and *dfrG-fos-lin*.

#### 3.3.2. Putative Resistance Phenotypes According to Beef Industries of Origin and the Sample Types

The frequency of putative resistance phenotypes detected in the 110 isolates of *L. innocua* consisted of trimethoprim, 8.2% (9/110), tetracycline, 33.6% (37/110), streptomycin, 0.9% (1/110), streptogramin, 0.9% (1/110), macrolide, 4.5% (5/110), lincosamide, 100% (110/110), fosfomycin, 100% (110/110), and erythromycin, 3.6% (4/110).

#### 3.3.3. Relationship between Phenotypic AMR Profile and Genomic AMR Gene

The data on the phenotypic AMR profile and genomic AMR gene profile are shown in Supplementary data, Table S5. A comparison of the phenotypic AMR profile detected in 16 antimicrobial agents with 13 genomic AMR genes revealed that only tetracycline had a high number (44) of phenotypic tetracycline-resistant isolates available for assessment. Furthermore, there was a wide disparity in the panel of phenotypic antimicrobial agents for comparison with the genomic AMR genes. The association between phenotypic and genomic AMR is as follows: phenotypic tetracycline (pTE) resistant-*tet*(*M*) gene positive, six (13.6%); p(TE) susceptible-*tet*(*M*) gene positive, three (6.8%); pTE resistance-*lin* gene positive, sixteen (36.4%); p(TE) resistance-*fosX* gene positive, eighteen (40.9%); and p(TE) resistance-*ImuG* gene positive, (2.3%). The differences in the frequencies were statistically significant (*p* < 0.001).

Overall, regarding the association between p(TE) resistance and the *tet*(*M*) gene, among the nine isolates, six (66.7%) were p(TE) resistant and carriers of the *tet*(*M*) gene, while three (33.3%) were p(TE) susceptible but positive for the *tet*(*M*) gene.

#### 3.3.4. Resistance and Virulence Genes across the Industries

**Figure 5:**
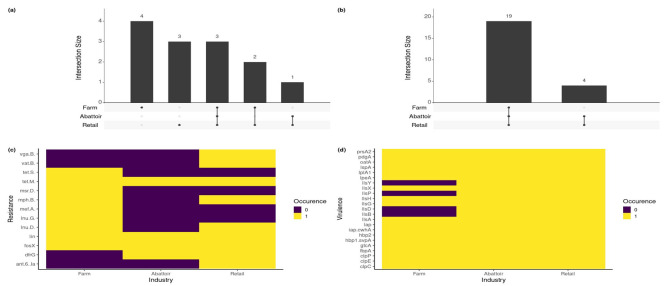
Resistance and virulence profiles across the three different industries. (**a**) Unique and shared resistance genes in the farm, abattoir, and retail industries. In farming environments, four unique AMR genes (lnuG, mefA, msrD, tetS) were present, compared with three (*ant.6.Ia, vatB, vgaB*) in the retail sector. These are represented by a black dot below the bar, with the number in the set on top of the bar. Three AMR genes (*fosX, lin, tetM*) were found in all three environments. (**b**) Shared virulence genes found in the three different industries. The three sectors shared a total of 19 virulence genes, with four (*llsB, llsD, llsP, llsY*) found exclusively in the abattoir and retail industries. (**c**,**d**) Presence/absence plots for the genes found in the three industries. Purple represents the absent, and yellow represents the present.

The resistance and virulence genes detected across the three industries are presented in Figure 5. In the farming sector, four unique AMR genes were detected, and three in the retail industry. The unique AMR genes in the farms were *lnu.G*, *mef.A*, *msr.D*, and *tet.S*, whereas *ant.6.Ia*, *vat.B*, and *vga.B* were exclusive to the retail sector. Shared by all three industries were *fosX*, *lin*, and *tet.M.* Farm and retail were found to share *lnu.D* and *mph.B*, with *dfrG* being the only AMR unique to both abattoirs and the retail industry. The farm, abattoir, and retail sectors displayed 19 virulence genes in common with four of the 23 virulence genes found exclusively in the abattoir and retail sectors (*llsB*, *llsD*, *llsP*, and *llsY*).

### 3.4. Occurrence of AMR Genes in L. innocua Isolates per Food and Sample Type

**Figure 6:**
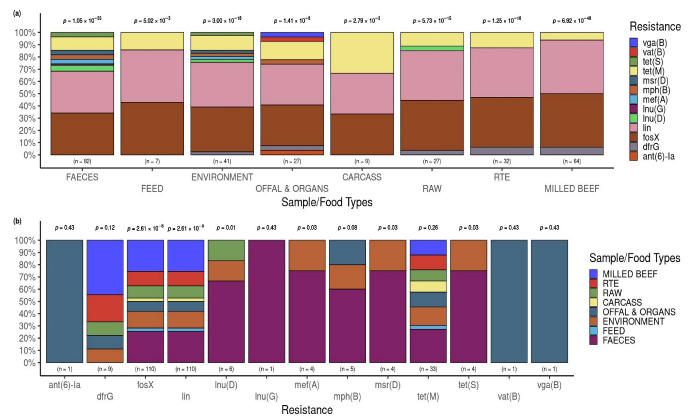
Occurrence of resistance genes according to the food and sample types. (**a**) Distribution of resistance genes across the food and sample types. The *p*-value associated with a proportion test within each food and sample type is indicated above the respective bar. (**b**) The food and sample types associated with each resistance gene detected. The *p*-values above each individual bar was obtained by proportion testing of the food and sample types associated with each resistance gene.

The *p*-values above the bars indicate a significant difference from the expected proportions for the resistance genes found within each food/sample type. The frequencies of AMR genes in *L. innocua* varied significantly (*p* < 0.05) for each of the eight sample/food types assessed (Figure 6a).

The *p*-values indicated above the bars revealed statistically significant (*p* < 0.05) differences from the expected proportions for the food/sample types within each AMR gene in only six: *fosX*, *lin*, *Inu*(*D*)**, *mef*(*A*)**, *msr*(*D*)**, and *tet*(*S*)** (Figure 6b).

### 3.5. Occurrence of Virulence Genes in L. innocua Isolates per Food and Sample Type

**Figure 7:**
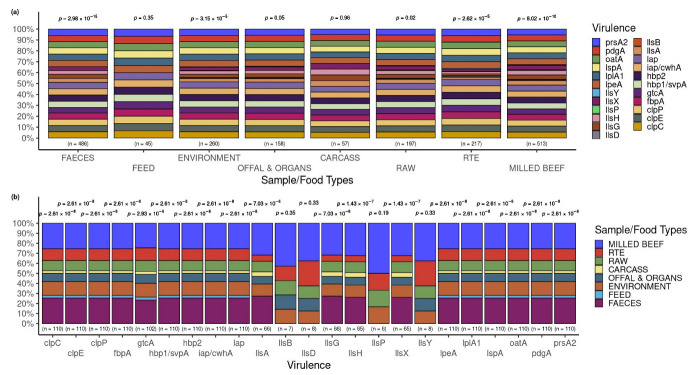
Occurrence of virulence genes according to food and sample types. (**a**) Distribution of virulence genes across the food and sample types. The *p*-value associated with a proportion test within each food and sample type is indicated above the respective bar. (**b**) The food and sample types associated with each virulence gene detected. The *p*-values above each individual bar was obtained by proportion testing of the food and sample types associated with each virulence gene.

The *p*-values above the bars indicate a significant difference from the expected proportions for the virulence genes detected within each food/sample type. The frequencies of virulence genes in *L. innocua* varied significantly (*p* < 0.05) for each of the eight sample/food types assessed (Figure 7a).

Within the 23 virulence genes observed, there were statistically significant (*p* < 0.05) differences from the expected proportions for the food/sample types within each of the 19 genes but not in *llsB* (*p* = 0.35), *llsD* (*p* = 0.33), *llsP* (*p* = 0.19), and *llsY* (*p* = 0.33) (Figure 7b).

## 4. Discussion

The current study is the first comprehensive study undertaken in South Africa on *L. innocua* recovered from three levels of cattle production (cattle farms), beef abattoirs (cattle slaughter), and beef/beef products retailing (retail outlets), concerning the genomic characterization of sequence types, resistance genes, and virulence genes. Both *L. monocytogenes* and *L. innocua* occupy the same niche in foods [11,12]; the detection of *L. innocua* indicates the possible presence of *L. monocytogenes* in foods. Unlike the present study, the few published genomic characterizations of *Listeria* species were studies conducted on *L. monocytogenes* strains recovered from the 2017–2018 large outbreak of human listeriosis [31,60], the report by Mafuna et al. [35] on the strains of *L. innocua* and *L. welshimeri* isolated from meat and food processing facilities in the country, and the sequencing of one isolate of *L. innocua* from a healthy goat [36]. The current study provides data on *L. innocua* in the country’s farm-abattoir-retail association. In other countries, *L. innocua* isolates recovered from meat are characterized using molecular methods [19,61].

It is interesting that in our study, the predominant STs of *L. innocua* detected differed significantly as to the source of isolates: ST637 (cattle farms), ST537 (abattoirs), and ST448 (retail outlets). This is in comparison to the *L. innocua* isolates obtained from retail outlets in Gauteng province, where nine STs were identified, of which ST448 (33.3%), ST1085 (23.3%), and ST637 (15%) were prevalent. Mafuna et al. [35] also identified nine STs, of which the most common were ST537 (56%) and ST1085. Also, only four STs (ST537, ST637, ST448, and ST1085) were common in both studies. The differences in the STs detected between both studies may be explained partly by the types of samples collected (beef versus meats), the source (retail outlets versus food processing facilities), and the number of locations (one province versus nine provinces). Reports by others have documented diversity in the STs, and their frequencies are affected by the geographical location, source, and types of samples from which the isolates originate, among other factors [20,32].

The industry sources (cattle farm, abattoir, and retail) of the *L. innocua* investigated had statistically significant effects on the overall detection frequency of *L. innocua* and, more importantly, the frequency of STs and AMR genes. This is evident from the findings across the three industries of the 12 STs detected in our study: statistically significant (*p* < 0.05), the highest frequency was observed for ST637 (cattle: 50%), ST448 (retail: 32.8%), ST537 (abattoir: 73.3%), ST1085 (retail: 21.3%), and ST1489 (11.5%). Similarly, the impact of the beef industry was demonstrated by our findings of significantly higher frequency on *tet*(*M*) (abattoir: 73.3%) and *dfrG* (retail: 13.1%). The differences in the distribution of STs and AMR genes in *L. innocua* across the three industries reflect the practices and activities at the three levels, thus affecting the spread and epidemiology of *L. innocua*, from cattle arms to abattoirs and finally to retail outlets. Failure to detect any significant effect of the beef industry on the frequency of carriage of virulence genes by *L. innocua* may be explained in part by the widespread high frequency of virulence genes, where the frequency ranged from 82.6% to 100% of 23 genes detected in 110 isolates recovered from the three industries.

In the current study, resistance genes *fosX* (100%), *lin* (100%), and *tet*(*M*) (30%) were predominantly detected. Similarly, Hanes and Huang [62] reported that in the USA, from 2010 through 2021, data analysis identified *fosX*, *lin*, *abc-f*, and *tet*(*M*) as the four most common AMR genes found in *L. monocytogenes.* Compared with published reports on resistance genes in *L. innocua*, the distribution of the resistance genes varied considerably [28,29].

In our study, it was important that there was a high variety of resistance genes detected in the isolates of *L. innocua* obtained from feedlots (61.5%) compared with the low diversity of resistance genes found in the isolates from communal farms. This is no surprise because animals at intensively managed feedlots receive cattle from diverse sources (farms and auctions) and mostly experience antibiotic pressure to control infections and disease. On the other hand, communal farms in South Africa rear fewer cattle (<10 per herd) in extensive or semi-intensive management systems with minimal antimicrobial agent use, often dictated by financial limitations posed to farmers by the cost of treatment.

The significantly higher diversity of AMR genes detected in *L. innocua* recovered from fecal and environmental samples may be explained partly by the fact that some of the fecal samples were pooled from around the feeding areas and environmental water and effluent samples; thus, a sample may have originated from several animals. Reports by others support our findings, where the frequency and distribution of resistance genes in *L. innocua* varied considerably by the types of samples from which the isolates originated [30,63].

Our investigation also revealed that the frequency of resistance genes was significantly associated with the STs of the *L. innocua* isolates in five STs: 637, 1482, 537, 1008, and 1489. It is also interesting to have detected ST-specific AMR genes, as demonstrated by the presence of gene *dfrG* only in *L. innocua* ST1489 and the fact that the four isolates that belonged to ST1610 were each carriers of multi-drug resistance (MDR) genes (*fosX*, *lin*, *inuD*, *metA*, *mph*, *msrD*, and *tetS*) in all four ST1610 isolates. The association of resistance genes with STs has been documented by others [64]. Regardless of the STs, it is of potential therapeutic significance that nine MDR genes were detected in our study, ranging from two to seven genes per isolate. Palaiodimou et al. [64] have also reported the occurrence of the MDR genes *bcrABC*, *emrC*, and *qacH* and emphasized the risk of AMR and MDR transfer to other bacteria, including *L. monocytogenes* [61,65].

Lincosamide and fosfomycin resistance genes, *linA* and *fosX*, were detected at a very high frequency of 100% each, indicating ubiquity in *L. innocua* genomes from this study. Our results are in line with a study by Ramadan et al. [66], which, using WGS analysis of *L. innocua* isolates, reported the presence of *fosX* in all the isolates assayed. Studies on *L. innocua* AMR are limited, but analysis of 1.696 *L. monocytogenes* isolates revealed the *fosX* gene to be part of the *Listeria* core genome, where all isolates harbored this gene [67]. The study also reported orthologs of *fosX* in *L. innocua*, another *Listeria* species. Furthermore, Parra-Flores et al. [68] reported that 100% of the strains of *L. monocytogenes* isolated from RTE foods in Chile of both genes, *fosX* (99.98%) and *lin* (97.8%), were detected in *L. monocytogenes* strains isolated during the period from 2010 through 2021 in the USA by Hanes & Huang [62]. Our findings, therefore, agree with the reports that *fosX* may be ubiquitous in *Listeria*.

Of the three predominant resistance genes (*fosX*, *lin*, and *tet*(*M*)), putative resistance to fosfomycin and tetracycline appears to be pertinent to South Africa because these antimicrobial agents are inexpensive, readily available, and used by farmers on livestock in the country [69]. However, tetracycline is the country’s most frequently used on livestock. Therefore, the detection of 30% of the *L. innocua* isolates recovered from the three levels of sampling (farm, abattoirs, and retail outlets) and the putative resistance encoded by the *tet*(*M*) and *tet*(*S*) genes based on WGS were 33.6%. Therefore, there is a potential for tetracycline-resistant *L. innocua* strains to enter the human food chain. It is relevant to mention that the prevalence of phenotypic tetracycline resistance exhibited by the same isolates of *L. innocua* using the disc diffusion method was 36.8% [37]. Interestingly, this phenotypic resistance correlates well with the putative resistance to tetracycline due to both the *tet*(*M*) and *tet(S*) genes, suggesting that the genes may have been partly responsible for the resistance detected. These findings suggest that tetracycline resistance may have been acquired with the potential for these antimicrobial genes to be transferred to commensal and pathogenic bacteria through the food chain, in addition to the fact that antimicrobial resistance in *L. monocytogenes* may have an adverse effect on the effective treatment of listeriosis in humans, as mentioned by Escolar et al. [61]. Studies have been reported on the resistance of bacterial pathogens, such as *E. coli*, *Salmonella*, and *Listeria* species, to tetracyclines in the livestock industry in South Africa [32,69]. The resistance of bacteria to tetracycline in South Africa has been attributed to the unregulated use of veterinary drugs, including tetracycline, in the country. This is attributed to the existing Fertilizers, Farm, and Agricultural and Stock Remedy Act (Act 36, 1947), which legalizes the use of certain antimicrobial agents, such as sulphonamides and trimethoprim, to be purchased over-the-counter, and they are used for treatment and as growth promoters [70]. Interestingly, the phenotypic resistance exhibited to tetracycline (36.8%) determined by Gana [37] correlates well with the putative resistance to tetracycline due to both *tet*(*M*) and *tet(S*) genes based on WGS on the same isolates, suggesting that the genes may have been partly responsible for the resistance detected. Other studies have similarly reported the correlation between phenotypic resistance and the carriage of corresponding encoding resistance genes [65,71,72]. However, a lack of correlation between these variables has also been reported by others [61]. It has been documented that bacteria may possess resistance genes but not express them, or they may be lost, thus limiting their application to their therapeutic implications and significance [73].

In our study, although forty-four *L. innocua* isolates were tested for phenotypic AMR and genomic AMR genes, only six (13.6%) were carriers of the *tet*(*M*) gene, while conversely, three (6.8%) of the TE-susceptible isolates were positive for the *tet*(*M*) gene. It was also interesting to have detected that 35 (79.5%) of the TE-resistant isolates of L. innocua were carriers of resistant genes (*fosX*, *lin*, and *InuD*) other than the *tet*(*M*) gene. These findings can be explained in part by the fact that the resistance genes in *Listeria* spp. and other bacteria may not be expressed [73,74,75]. Furthermore, our findings that approximately 80% of the TE-resistant isolates were carriers of the two predominant genes (*FosX* and *lin* genes) were detected. Therefore, the resistance encoded by these two genes was not assayed in the disc diffusion method, and the predominant resistance genes observed in our study were not tested phenotypically in the earlier study. The therapeutic significance of these findings cannot, therefore, be ignored and requires further investigation.

*L. innocua* is considered non-pathogenic. Previous analyses have suggested that *L. monocytogenes* and *L. innocua* evolved from a common virulent ancestor. During evolution, consecutive losses of virulene genes critical to host adaptation were associated with the emergence of *L. innocua* [15]. Rare, atypical *L. innocua* strains that harbor LIPI-1 and *inlA* and are hemolytic and weakly virulent may represent an intermediary evolutionary stage. In addition, rare, atypical *L. monocytogenes* strains resulted from the spontaneous loss of virulence genes and were nonhemolytic [75]. In the present study, 23 virulence factors were detected in the 110 isolates of *L. innocua* using WGS, thus providing a spectrum of the virulence factors carried by the isolates, unlike PCR, which provided information specific only to the primers targeted [76,77]. Unlike *L. innocua*, the ability of *L. monocytogenes* to cause listeriosis is known to be multifaceted and has been attributed to six virulence genes, *prfA*, *plcA*, *hly*, *mpl*, *actA*, and *plcB*, which are located in the PrfA-dependent virulent gene cluster known as LIPI-1 [77,78], other *Listeria* pathogenicity islands, namely LIPI-3 and LIPI-4, Internalins (*inl*) genes, and other virulence genes, as reported by Glimour et al. [79]. None of our *L. innocua* isolates contained virulence genes and are therefore classified as non-pathogenic [80]. However, it has emerged that some strains of *L. innocua* have been demonstrated to contain virulence genes that have contributed to their weak virulence [15]. Some of the factors documented by others in strains of *L. innocua* include the carriage of virulence factors such as LGI2, LGI3, LIPI-3, and LIPI-4 [14,35].

It was noteworthy to have detected a broad spectrum of virulence genes and groupings based on their STs. According to the STs, this distribution of virulence genes has been documented in *L. monocytogenes*, where some are more associated with listeriosis depending on the virulence genes they carry, as reported in a recent outbreak of human listeriosis caused by *L. monocytogenes*, ST6 [24,35]. Notwithstanding the high frequency of virulence genes in *L. innocua* isolates recovered from the three industries of beef production in South Africa, it is important to note that the presence/absence of virulence genes in *L. monocytogenes* was not a predictor of the virulence potential of *L. monocytogenes* [81]. Similarly, we should interpret the presence of virulence genes in *L. innocua* with caution. Further assessments, including hemolytic and virulence assays, on our *L. innocua* strains are needed.

Our analysis of the occurrence of AMR and virulence genes, regardless of the industry sources of the isolates of *L. innocua*, revealed the significant occurrence of AMR and virulence genes in most of the food and sample types assessed. Similar findings have been reported by others [1,35]. These findings are indicative that the consumption of certain food types may increase exposure to AMR-carrying *L. innocua* strains with potential transfer to pathogenic *L. monocytogenes* in the same food niche, thus posing therapeutic implications [61,65].

## 5. Conclusions

For the first time in South Africa, this study provided a comprehensive genomic characterization of resistance and virulence genes in *L. innocua* isolated from three levels (production, processing, and retailing) of the beef industry using WGS. The MSTs using the profiles of the STs, AMRs, and virulence genes revealed a diversity in their spread and clustering regardless of the sources and sample types from which the *L. innocua* isolates originated. It is also important that at each of the three industries (cattle farms, abattoirs, and retail), significant effects were detected on the frequency of five STs and two AMR genes, thus providing evidence of their potential epidemiological importance. Furthermore, the phylogenies based on 120 GTDB core bacterial marker genes confirmed the genetic relatedness of the *L. innocua* isolates. This was shown by the clustering of isolates originating from abattoirs as well as those from communal and cow-calf operations (farm level). The high frequency of resistance genes *tet*(*M*) and *fosX* observed in this study suggests that the use of tetracycline and fosfomycin in the livestock industry in the country and their role in the development of bacterial antimicrobial resistance should be reviewed. This is particularly relevant because a comparison of the phenotypic AMR with the genomic AMR genes revealed that approximately 80% of TE-resistant isolates, although negative for the *tet*(*M*) gene, were carriers of the *fosX* and *lin* genes. It is, therefore, imperative to phenotypically determine the resistance they encode. Finally, caution is needed in extrapolating the data based on the presence and absence of genes to the potential phenotype (i.e., resistance and virulence potential). The study has provided invaluable data on the status of *L. innocua* in the cattle industry food chain in the country.

## Figures and Tables

**Table 1:** Characteristics of *L. innocua* isolates according to the three industries (farm, abattoir, and retail).

	Sample		Isolates of *L. innocua*														
	No. of Samples	No. (%) Positive	No. of Isolates	No. (%) of Isolates That Belong to ST ^a^:													
Industry	Tested	For *L. innocua*	T\ested	ST637	ST448	ST537	ST1085	ST1087	ST1489	ST1482	ST1008	ST602	ST43	ST 1619	ST 1610	ST3007	ST 2754
Cattle farms ^b^	328	37 (11.3)	34	17 (50.0)	2 (5.9)	2 (5.9)	1 (2.9)	1 (2.9)	0 (0.0)	6 (17.6)	0 (0.0)	0 (0.0)	1 (2.9)	0 (0.0)	4 (11.8)	0 (0.0)	1 (2.9)
Abattoirs ^c^	262	15 (5.7)	15	2 (13.3)	0 (0.0)	11 (73.3)	0 (0.0)	0 (0.0)	1 (6.7)	0 (0.0)	0 (0.0)	0 (0.0)	0 (0.0)	0 (0.0)	0 (0.0)	0 (0.0)	0 (0.0)
Retail outlets ^d^	400	65 (16.3)	61	10 (16.4)	20 (32.8)	2 (3.3)	13 (21.3)	0 (0.0)	7 (11.5)	1 (1.6)	5 (8.2)	1 (1.6)	0 (0.0)	1 (1.6)	0 (0.0)	1 (1.6)	0 (0.0)
Total	990	117 (11.8)	110	29 (26.4)	22 (20.0)	15 (13.6)	14 (12.7)	1 (0.9)	8 (7.3)	7 (6.4)	5 (4.5)	1 (0.9)	1 (0.9)	1 (0.9)	4 (3.6)	1 (0.9)	1 (0.9)
	No. of samples	No. (%) positive	No. of isolates	No (%) positive for AMR gene ^e^													
Industry	tested	*for L. innocua*	tested	*fosX*	*Lin*	*tet* (*M*)	*dfrG7*	imuD	ImuG	mefA	mph(B)	mrs(D)	tet(S)	vatB	vga	ant.6.1a	
Cattle farms ^b^	328	37 (11.3)	34	34 (100.0)	34 (100.0)	7 (20.6)	0 (0.0)	5 (14.7)	1 (2.9)	4 (11.8)	4 (11.8)	4 (11.8)	4 (11.8)	0 (0.0)	0 (0.0)	0 (0.0)	
Abattoirs ^c^	262	15 (5.7)	15	15 (100.0)	15 (100.0)	11 (73.3)	1 (6.7)	0 (0.0)	0 (0.0)	0 (0.0)	0 (0.0)	0 (0.0)	0 (0.0)	0 (0.0)	0 (0.0)	0 (0.0)	
Retail outlets ^d^	400	65 (16.3)	61	61 (100.0)	61 (100.0)	15 (24.6)	8 (13.1)	1 (1.6)	0 (0.0)	0 (0.0)	1 (1.6)	0 (0.0)	0 (0.0)	1 (1.6)	1 (1.6)	1 (1.6)	
Total	990	117 (11.8)	110	110 (100.0)	110 (100.0)	33 (30.0)	9 (8.2)	6 (5.5)	1 (0.9)	4 (3.6)	5 (4.5)	4 (3.6)	4 (3.6)	1 (0.9)	1 (0.9)	1 (0.9)	

^a^ Two isolates (one each from a farm and a retail outlet) could not be assigned to any ST; four STs (ST619, 602, 108, 43). ^b^ Comprising communal farms (n = 10; eight isolates), cow-calf farms (n = 10; fourteen isolates), and feedlots (n = 3; twelve isolates). ^c^ Abattoirs consisting of high-throughput (n = 6; twelve isolates), low-throughput, and LT (n = 2; three isolates). ^d^ Retail outlets made up of chain outlets (n = 30; sixteen isolates), large (n = 10; seventeen isolates), medium (n = 6; eighteen isolates), and small (n = 2; ten isolates). ^e^ Four resistance genes were detected in one isolate each: ant.6.1a (retail outlet), *inuG* (*farm*), *vatB* (retail outlet), and *vga* (retail outlet).

**Table 2:** Distribution of AMR gene patterns among *Listeria innocua* by source of isolates.

Number of Resistance		No. (%) of *L. innocua* Isolates with Resistance Genes by Source of Samples				Total (No., %),
Genes ^a^ (No. of Patterns)	Resistance Gene Pattern	Cattle Farm (n = 34) ^c^	Beef Abattoirs (n = 15) ^d^	Retail Outlets (n = 61) ^e^	*p*-Value	n = 110
2 (1)	*^b^ fosX-lin*	23 (67.6)	3 (20.0)	38 (62.3)	0.0048	64 (58.2)
3 (2)	*fosX-lin-tet*(*M*)	5 (14.7)	11 (73.3)	11 (18.0)	0.00001	27 (24.5)
	*dfrG-fosX-lin*	0 (0.0)	1 (6.7)	7 (11.4)	0.0474	8 (7.3)
4 (4)	*fosX-lin-Inu*(*D*)*-tet*(*M*)	1 (2.9)	0 (0.0)	1 (1.6)	1	2 (1.8)
	*fosX-lin-Inu*(*G*)*-tet*(*M*)	1 (2.9)	0 (0.0)	0 (0.0)	0.7539	1 (0.9)
	*fosX-lin-tet*(*M*)*-vat*(*B*)**	0 (0.0)	0 (0.0)	1 (1.6)	1	1 (0.9)
	*dfrG-fosX-lin-tet*(*M*)	0 (0.0)	0 (0.0)	1 (1.6)	1	1 (0.9)
5 (2)	*ant*(*6*)*-Ia-fosX-lin-mph*(*B*)*-tet*(*M*)	0 (0.0)	0 (0.0)	1 (1.6)	1	1 (0.9)
	*fosX-lin-tet*(*M*)*-vat*(*B*)*-vga*(*B*)**	0 (0.0)	0 (0.0)	1 (1.6)	1	1 (0.9)
7 (1)	*fosX-lin-Inu*(*D*)*-mef*(*A*)*-mph*(*B*)*-msr*(*D*)*, tet*(*S*)**	4 (11.8)	0 (0.0)	0 (0.0)	0.146	4 (3.6)

^a^ Overall, 13 resistance genes were detected using WGS. ^b^ Resistance genes (antimicrobial class): *fosX* (phosphonic acid), *lin* (lincosamide), *tet*(*M*) (tetracycline), *dfrG* (diaminopyrimidine), *Inu*(*D*)** (lincosamide), *inu*(*G*) (lincosamide), *vat*(*B*) (streptogramin), *ant*(6)*-Ia* (aminoglycoside), *mph*(*B*) (macrolide), *vga*(*B*)** (streptogramin), *inu*(*D*) (lincosamide), *mef*(*A*) (streptogramin), *mph*(*B*) (macrolide), *msr*(*D*) (streptogramin), and *tet*(*S*) (streptomycin). ^c^ Recovered from samples of feces, feed, and the environment from cattle farms (communal, cow-calf, and feedlot). ^d^ Comprised isolates obtained from samples of carcass swabs and the environment collected from beef abattoirs (high- and low-throughput). ^e^ Isolates recovered from raw beef, milled beef, ready-to-eat beef, and offal and organs sampled from retail outlets (chain, large, medium, and small).

## Data Availability

All samples have been deposited under NCBI BioProject: PRJNA215355 and can be searched based on the isolate CFSAN identifier.

## Supplementary Materials

The following are available online at https://www.mdpi.com/article/10.3390/pathogens12081062/s1, Table S1. Coverage of the sequences used in the study, Table S2. Sources, types of samples, sequence types, and resistance genes detected in 110 *L. innocua*, Table S3. Sources, types of samples, sequence types, and virulence genes detected in *L. innocua* isolates, Table S4. Sequence Type (ST) profiles and associated loci, Table S5. Sources, types of samples, sequence types, phenotypic AMR, and genomic AMR profiles, types of samples, sequence types, phenotypic AMR and genomic AMR profiles.
